# Screening and characterization of amylase and cellulase activities in psychrotolerant yeasts

**DOI:** 10.1186/s12866-016-0640-8

**Published:** 2016-02-19

**Authors:** Mario Carrasco, Pablo Villarreal, Salvador Barahona, Jennifer Alcaíno, Víctor Cifuentes, Marcelo Baeza

**Affiliations:** Departamento de Ciencias Ecológicas, Facultad de Ciencias, Universidad de Chile, Las Palmeras 3425, Casilla 653, Santiago, Chile

**Keywords:** Amylase, Cellulase, Cold-active enzymes, Psychrotolerant yeasts

## Abstract

**Background:**

Amylases and cellulases have great potential for application in industries such as food, detergent, laundry, textile, baking and biofuels. A common requirement in these fields is to reduce the temperatures of the processes, leading to a continuous search for microorganisms that secrete cold-active amylases and cellulases. Psychrotolerant yeasts are good candidates because they inhabit cold-environments. In this work, we analyzed the ability of yeasts isolated from the Antarctic region to grow on starch or carboxymethylcellulose, and their potential extracellular amylases and cellulases.

**Result:**

All tested yeasts were able to grow with soluble starch or carboxymethylcellulose as the sole carbon source; however, not all of them produced ethanol by fermentation of these carbon sources. For the majority of the yeast species, the extracellular amylase or cellulase activity was higher when cultured in medium supplemented with glucose rather than with soluble starch or carboxymethylcellulose. Additionally, higher amylase activities were observed when tested at pH 5.4 and 6.2, and at 30–37 °C, except for *Rhodotorula glacialis* that showed elevated activity at 10–22 °C. In general, cellulase activity was high until pH 6.2 and between 22–37 °C, while the sample from *Mrakia blollopis* showed high activity at 4–22 °C. Peptide mass fingerprinting analysis of a potential amylase from *Tetracladium* sp. of about 70 kDa, showed several peptides with positive matches with glucoamylases from other fungi.

**Conclusions:**

Almost all yeast species showed extracellular amylase or cellulase activity, and an inducing effect by the respective substrate was observed in a minor number of yeasts. These enzymatic activities were higher at 30 °C in most yeast, with highest amylase and cellulase activity in *Tetracladium* sp. and *M. gelida,* respectively. However, *Rh. glacialis* and *M. blollopis* displayed high amylase or cellulase activity, respectively, under 22 °C. In this sense, these yeasts are interesting candidates for industrial processes that require lower temperatures.

**Electronic supplementary material:**

The online version of this article (doi:10.1186/s12866-016-0640-8) contains supplementary material, which is available to authorized users.

## Background

Yeasts inhabit almost all environments on the earth including cold environments in which they are permanently exposed to temperatures below 5 °C. These cold-adapted yeasts are classified as psychrophilic if their optimum and maximum temperatures for growth are ≤ 15 °C and ≤ 20 °C, respectively, or as psychrotolerant (psychrotrophic) if their maximum temperature for growth is above 20 °C [1, 2]. An adaptation of yeasts to cold is the production of extracellular hydrolytic enzymes to use the available carbon sources and in this way they contribute to nutrient recycling and organic matter mineralization [3, 4]. The main adaptation of enzymes from psychrophiles is the maximization of their flexibility resulting in a slower decrease of the reaction rate when temperature is decreased in contrast to their mesophile or thermophile counterparts [5]. Psychrophilic enzymes have higher enzymatic activities at lower temperatures than their mesophyll equivalents [6] and a reduced thermal stability to counteract freezing [7]. These features have attracted the attention of many scientists in recent years due their potential for application in industry. The production of cold-active enzymes including amylase, lipase, protease, cellulase, pectinase and esterase has been reported in yeast species and generally the properties of yeast enzymes differ from those produced by other microorganisms [8, 9]. Cold active amylases and cellulases have great potential to be applied in processes that may require low temperatures such as those in the food, biofuels and detergents industries [10, 11]. Amylolytic enzymes are comprised of three main sub-groups: α-amylase, β-amylase and glucoamylase [12, 13]. Although all of these enzymes are able to hydrolyze α-glucosidic bonds in starch, they have structural and catalytic differences [14]. The complete degradation of raw starch is fundamental to the microbial industrial production of biofuels, and is currently accomplished by supplementation of α-amylase and glucoamylase during the fermentative processes [15].

Cellulases are responsible for the hydrolysis of cellulose to sugars in the environment, and cellulose is the largest source of renewable energy on the planet [16]. Cellulases are produced by bacteria and fungi [17–19], and these enzymes are useful in food industry, environmental remediation, fuel production and particularly in the laundry industry [16]. The most commonly used cellulases in industry are produced by fungi, which have optimum temperature at 50 °C [20]. Although cellulases from some psychrophilic yeasts have been described, within cold active enzymes, cellulases remain less studied [21].

To achieve a cost-effective process and to avoid the production of undesirable by-products generated at higher temperatures, a current research effort is aimed at searching for microorganisms that secrete amylases or cellulases that are active at lower temperatures. These microorganisms are even more appealing if they use economical culture media containing raw starch or cellulose as the main constituents [22–25]. Previously, our group isolated yeasts from soil samples from the sub-Antarctic region belonging to 22 species, and in preliminary assays several of them showed amylase and cellulase activities, respectively [26]. In this work, these yeasts were further characterized with respect to their assimilation and fermentation of soluble starch (SS) and carboxymethylcellulose (CMC). The enzymatic activities in extracellular protein samples obtained from yeasts cultivated in different media were evaluated at different pH and temperatures. Protein samples from yeast species with major amylase or cellulase activities were fractioned by ammonium sulfate precipitation and the protein fractions having the activity of interest were analyzed by peptide mass fingerprint, obtaining promising results.

## Results and discussion

### Growth and fermentation assays

**Fig. 1:**
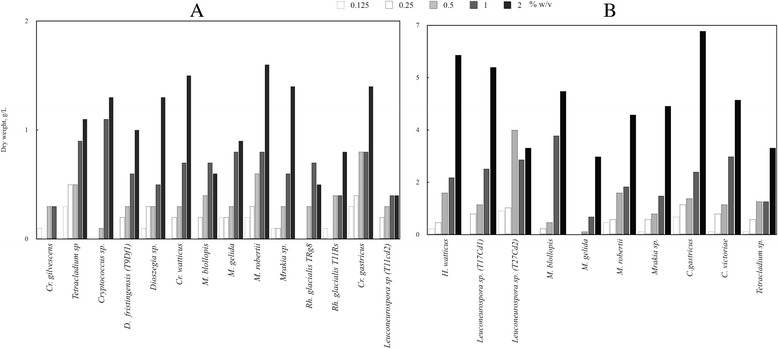
Biomass reached by the analyzed yeast species cultured with different carbon sources. Yeasts were cultured in minimal YNB medium supplemented with SS (**a**) or CMC (**b**) until the stationary phase of growth. The concentration (% w/v) of each carbon source is indicated at the top of graphs. The basal growth of yeasts in YNB without the supplementation of the carbon sources was subtracted in each case

Yeasts that previously showed positive results for amylase and cellulase activities in colony assays were analyzed for their ability to grow in minimal media (YNB) with SS or CMC as the sole carbon source. As shown in Fig. 1, all tested yeasts were able to grow on these media. In media supplemented with SS, the majority of yeast species produced more biomass at 2 % than at lower concentrations, except *Cryptococcus gilvescens* and *Rh. glacialis* B19 that reached a higher biomass when cultured with 0.5–1 and 1 % of SS, respectively (Fig. 1a). With the exception of *Leuconeurospora* sp. (T27Cd2), which reached more biomass when cultured with 0.5 % CMC, all yeasts showed a higher biomass when cultured with 2 % CMC than with lower concentrations. The ability of the yeast to perform alcoholic fermentation using both compounds as carbon sources was evaluated by the Durham method and ethanol quantification in liquid cultures, but negative results were obtained for all yeast species (not shown). In the case of glucose, positive results were obtained for *Candida sake*, *Mrakia blollopis*, *Mrakia gelida*, *Mrakia robertii* and *Wickermanomyces anomalus*. Due to an increase in the viscosity of the medium, the maximum concentration of SS or CMC that could be added to culture media was 2 %, so it was not possible to test higher concentrations. It is important to mention that a significant decrease of the medium viscosity was observed concomitantly with yeast growth. Taken together, these results suggest that the analyzed yeast species are able to assimilate SS and/or CMC, but they are not able to ferment these compounds.

### Enzyme activities of extracellular protein samples

**Fig. 2:**
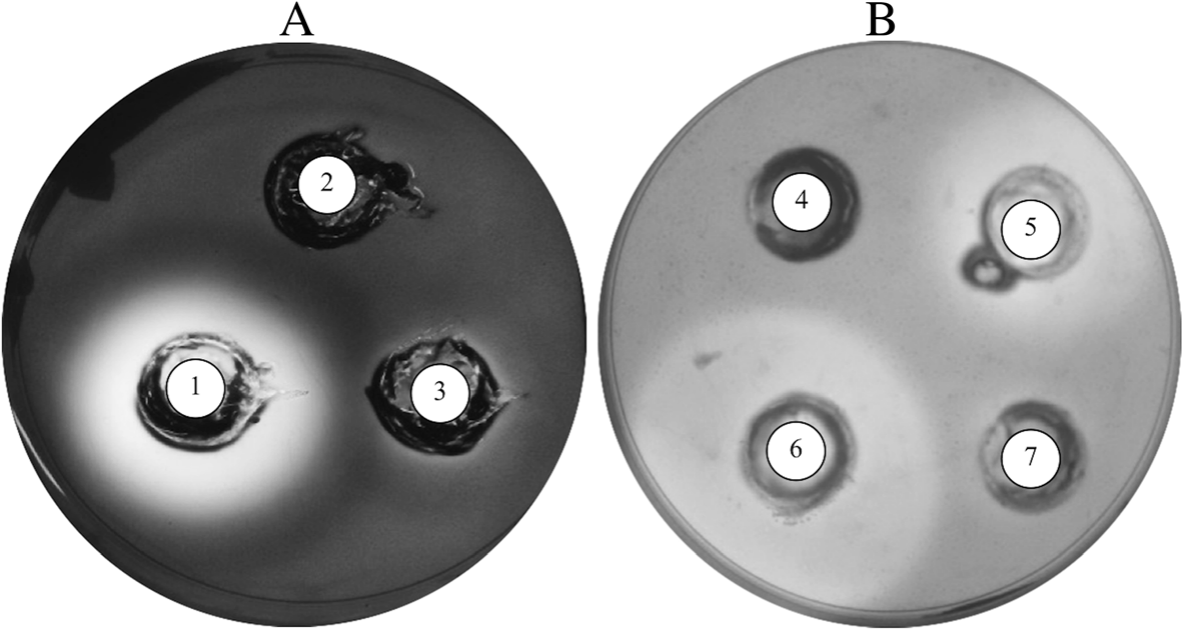
Enzymatic activities determined by well test assays. Extracellular protein samples were deposited into wells generated in the plates to analyze amylase (**a**) or cellulase (**b**) activities, which were incubated at 15 °C. Protein samples in wells: 1, *D. fristingensis* (T9Df1); 2, *D. fristingensis* (T9Df1) inactivated at 100 °C for 15 min; 3, *Leuconeurospora* sp.; 4, *Cr. gastricus*; 5, *D. fristingensis* (T11Df1); 6, *L. fragaria*; 7, *W. anomalus*

**Fig. 3:**
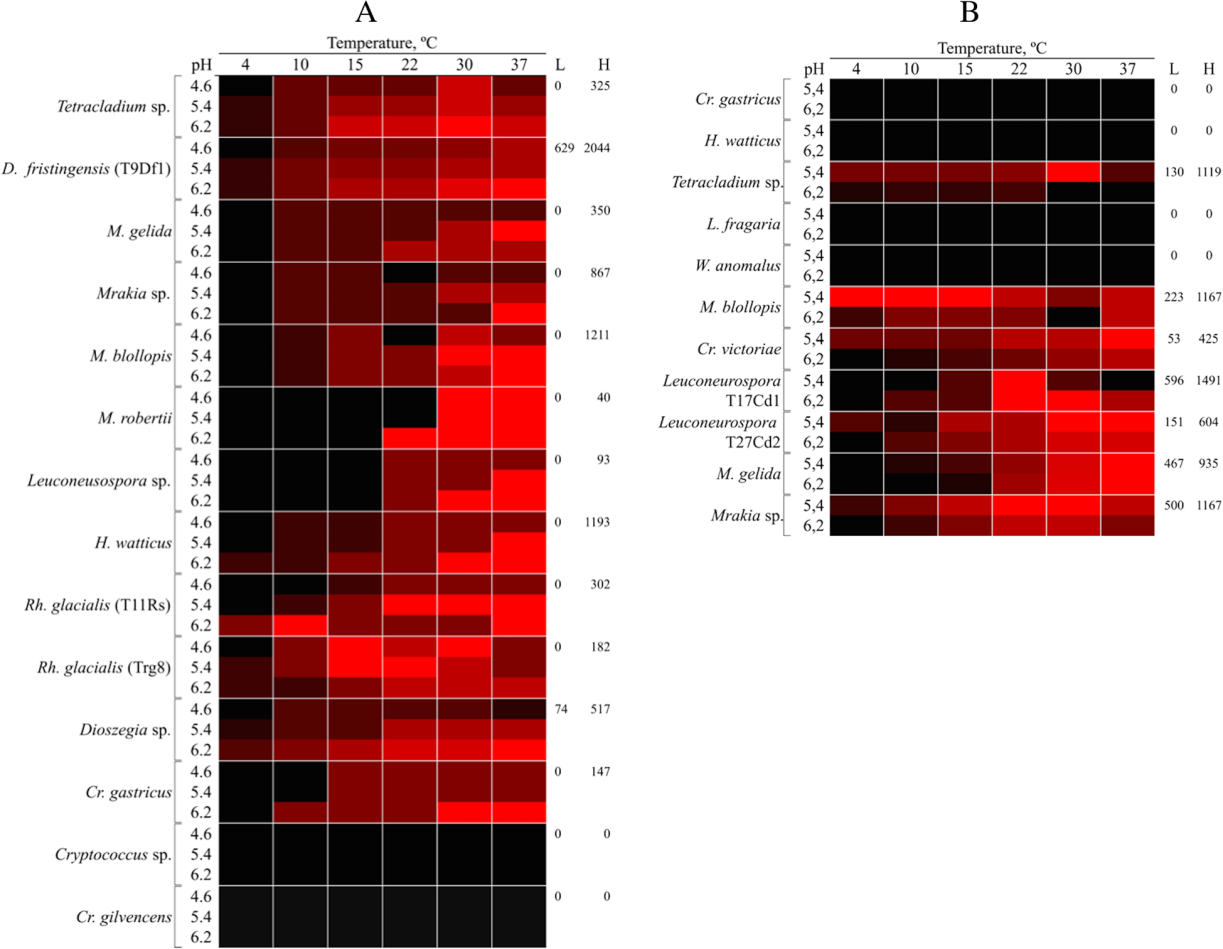
Enzyme activities in extracellular protein samples. Total proteins were obtained from cell-free supernatants of the analyzed yeast species cultured in YM media supplemented with glucose. The amylase (**a**) and cellulase (**b**) activity was evaluated by the well test assay at different pH and temperatures, and normalized by the total protein amount (mm/mg total protein). The gradient color scale indicated in the figure represents the lowest (L, black) to the highest (H, red) activity recorded values from culture supernatant of each yeast species

**Fig. 4:**
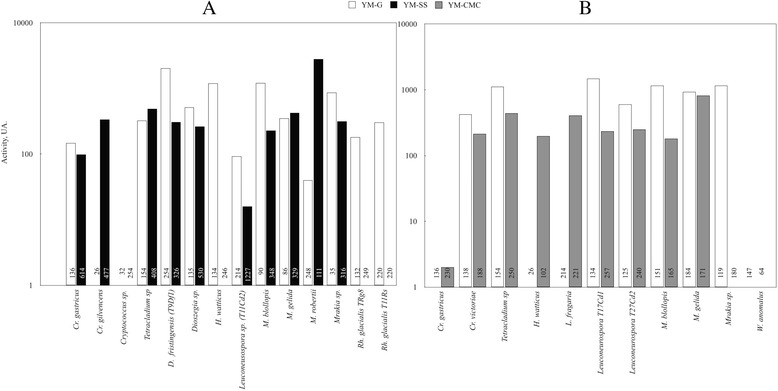
Comparison of extracellular enzymatic activities. Protein samples were obtained from yeast cultures in YM medium supplemented with glucose (YM-G), SS (YM-SS) or CMC (YM-CMC). The amylase (**a**) and cellulase (**b**) activities were evaluated and normalized by total protein amount (μg) used in the assays, which is indicated in each column

Total secreted proteins were obtained by ammonium sulfate precipitation from supernatants from yeast cultures using YM medium supplemented with glucose, and enzyme activities in the samples were evaluated by well test assays. In all assays, denaturalized protein controls corresponding to the protein sample incubated at 100 °C for 15 min were included and in all cases, no activity was observed, as shown in well 2 from Fig. 2a. As exemplified in Fig. 2, a high amylase activity was observed in *D. fristingensis* (T9) samples and a lower activity was observed in samples from *Leuconeurospora sp.* T11cd2 cultures (Fig. 2a). *Le. fragaria* and *Mrakia sp*. culture samples showed higher cellulase activities (Fig. 2b). In addition, enzyme activities of the protein samples were tested at different pH and temperature conditions including pH 4.6, 5.4 and 6.2; and 4, 10, 15, 22, 30 and 37 °C. The protein samples from the majority of the yeasts analyzed had higher amylase activities at pH 5.4 and 6.2, and at 30 and 37 °C (Fig. 3a). The highest amylase activities were observed in protein samples from *Dioszegia fristingensis* (T9Df1), *M. blollopis, Holtermaniella watticus* and *Tetracladium* sp., which were higher at pH 6.2 and 37 °C. No amylase activity was observed in samples from *Cryptococcus sp*. and *Cr. gilvescens*. Cellulase activity could not be assayed at pH 4.6 because the medium in the agar-plates did not solidify at this pH. As shown in Fig. 3b, the highest cellulase activity was observed in samples from *Leuconeurospora* T17Cd1 at pH 6.2 and 22–30 °C. Also, high activities were observed in samples from *Mrakia* sp*.* (pH 5.4; 22–30 °C), *M. blollopis* (pH 5.4; 4–15 °C) and *Tetracladium* sp. (pH 5.4; 30 °C). No cellulase activity was observed in samples from *Cr. gastricus*, *H. watticus*, *Leucosporidiella fragaria* and *Wickermanomyces anomalus* in the assayed conditions. In general, the halos observed in the enzyme activity assays had variable degrees of turbidity. However, samples from *Tetracladium* sp. and *M. gelida* gave very clear halos in the amylase and cellulase activity assays, respectively, suggesting greater substrate degradation. Although all yeasts were able to grow in medium with SS or CMC as the sole carbon source, the corresponding extracellular enzyme activity was not observed in some of the protein samples as mentioned above. Considering that it has been previously reported that CMC has an inducing effect in the cellulase activity from the fungus *Ganoderma applanatum* MR-56 [27] and that amylolytic activity was induced by SS in bacterial isolates [28], it is possible that the yeasts tested in this work require the presence of the substrate to induce the expression of the corresponding genes that encode the respective enzyme activity. To evaluate this possibility, the yeasts were cultured in YM medium supplemented with SS or CMC, the extracellular proteins were obtained and the activities measured (Additional file 1). Figure 4 shows the enzyme activities of protein samples from yeast cultivated in medium supplemented with glucose, SS or CMC, at the pH and temperature at which the highest activity was observed in each case. An amylase activity inducing effect by SS was observed only in samples obtained from four yeast species, most notably in *Cr. gilvescen*s and *M. robertii* (Fig. 4a). In the other species, the amylase activity was higher in samples from yeast cultivated in medium supplemented with glucose; even more for *H. watticus*, *Rh. glacialis* T8Rg and *Rh. glacialis* T11Rg, no amylase activity was detected when the yeasts were grown in media supplemented with SS. For *Cryptococcus* sp., no amylase activity was observed in the protein samples obtained from cultures in both conditions. In the analyses of cellulase activities, an inductor effect by CMC was observed only in samples from *Cr. gastricus, H. watticus* and *Le. fragaria*, whereas in the samples from the other yeasts species, the highest cellulase activities were observed when yeasts were cultivated in medium supplemented with glucose (Fig. 4b). No cellulase activity was observed in samples from *W. anomalus* grown in either condition. As indicated in each column of Fig. 4, for almost all yeast species, the total protein amounts were higher in samples from cultures supplemented with SS or CMC than with glucose; however, this increase in total protein content was not correlated with an increase of either amylase or cellulase activity in the samples. Although *Cryptococcus sp*. and *W. anomalus* were able to grow using SS and CMC as the sole carbon source, and amylase and cellulase activities were previously detected in yeast colony assays, neither activity was detected in extracellular protein samples from yeast cultures in either condition. It is possible that the corresponding enzymes are anchored to the cell wall, as this has been described for amylase-like enzymes in two species of *Aspergillus* [29, 30]. There is limited data regarding amylase activity in the yeast genera described in this work; amylase activity was described in *Cryptococcus sp*. [31] and *Cr. flavus* [32], the latter of which was successfully expressed in *S. cerevisiae* [33], *Mrakia blollopis* [34], *Rhodotorula svalbardensis sp*. nov [35] and *Tetracladium setigerum* [36]. No reported data were found in literature about amylase activity in the genera *Dioszegia*, *Leuconeurospora* and *Holtermaniella*. Cellulase activity has been described in *Cryptococcus laurentii* and *Cryptococcus nemorosus* [37], *Tetracladium* [36], and in *Mrakia* species isolated from Arctic puddles [38]. No data were found in the literature regarding cellulase activity for the genera *Dioszegia*, *Leuconeurospora*, *Leucosporidiella* and *Wickermamomyces*. The yeast studied in this work were originally isolated from soil samples of King George Island [26] in which there is a considerable input of organic materials, including vegetation. Furthermore, the temperatures in this region are warmer than the rest of Antarctica, having seasonal changes and that can reach up to 20 °C in summer [26, 39–41]. According to this, the yeasts studied that showed extracellular cellulase and amylase activities, can use the complex carbon sources, suggesting an active role in vegetable residues recycling and mineralization in this cold environment. Furthermore, the amylase and cellulase activities were detected in a wide range of temperature (4 to 30 °C) and the majority showed higher activities at moderate temperatures (22 or 30 °C). However, these temperatures are lower than those described for these enzymes in mesophilic organisms, which generally show optimal activities at temperatures over 40 °C [42, 43]. This last point reflects the adaptations of the yeasts studied here to the conditions of the sub-Antarctic region where they thrive.

### Identification of proteins

**Fig. 5:**
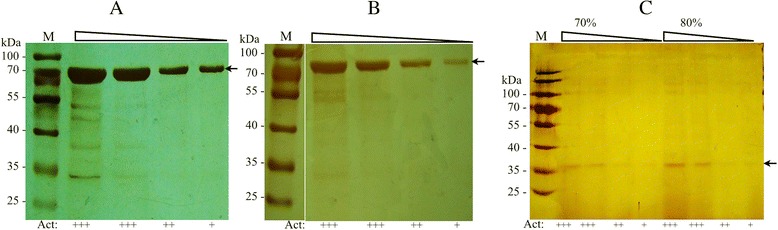
Extracellular proteins analysis by SDS-PAGE. Proteins samples were obtained from yeast cultures of *Tetracladium* sp. (**a** and **b**) and *M. gelida* (**c**) in YM medium supplemented with glucose (**a** and **c**) or soluble starch (**b**). Samples were fractioned with ammonium sulfate. The 60 % fraction, which had the highest amylase activity (**a** and **b**), and the 70 and 80 % fractions (**c**) that had the highest cellulase activity are shown. Serial dilutions of the samples were made (indicated at the top of gels) and the degree of the determined amylase or cellulase activity by the well assay is indicated at the bottom of each gel lane as follows: highest (+++) and lowest (+) recorded activity. The arrows indicate the protein band associated to amylase (**a** and **b**) or cellulase activity (**c**). M, protein marker

Taking into account that currently the industrial production of biofuels is accomplished by supplementation of amylases during the fermentative processes, which is mainly carried out by *S. cerevisiae* [15], it is desirable that the used enzymes have high activity at temperatures near to 30 °C. This was the case of *Tetracladium sp*. and *M. gelida* for amylase and cellulase activity, respectively, so they were selected for further analyses. The yeast *Tetracladium sp*. was cultured in liquid YM media supplemented with glucose or SS, and the extracellular proteins were fractionated by precipitation with ammonium sulfate (20 to 80 %). Amylase activity was determined in each fraction, revealing that the fraction obtained with 60 % of ammonium sulfate had the highest activity, so this condition was used for the subsequent analyses. Protein samples obtained from cultures supplemented with glucose or SS (Fig. 5a) had a main protein band of about 70 kDa, which after serial dilutions was the only band observed in SDS-PAGE concomitantly with amylase activity. Similar analysis was performed for the yeast *M. gelida* that showed cellulase activity, which was detected in the fractions of 70 and 80 % of ammonium sulfate from cultures supplemented with glucose and CMC. In analyses of samples from cultures with glucose, a main protein band of about 36 kDa was observed in samples with cellulase activity (Fig. 5b). Although we made several attempts to characterize the extracellular proteins from *M. gelida* grown in the presence of CMC, the quality of these results was always poor. As in previously reported results [44], this may be due to remaining CMC in the culture supernatant that interfered with the precipitation of proteins and SDS-PAGE analysis protocols, despite performing extensive dialysis of samples. The described 70 and 36 kDa protein bands were analyzed by peptide mass fingerprinting, and the results were analyzed using the Mascot search engine. In the case of the probable cellulase protein, no significant matches were found in the database. For the probable amylase protein, several peptides matched completely with fungal glucoamylases whose sizes ranged from 58 to 68 kDa, which is in accordance with the calculated size of the protein band mentioned above. The peptides ALVEGSTFASKVGASCSWCDSQAPQVLCFLQR, GSSFFTIAVQHR, CDSQAPQVLCFLQR, QAPQVLCFLQR and APQVLCFLQR, matched with glucoamylase precursor protein (XP_001213553) of *Aspergillus terreus* (NIH2624); the peptide DAALTMK with glucoamylase (ENH88038) of *Colletotrichum orbiculare* MAFF 240422; the peptides DAALTMK and SQAVIQTANNPSGSLLPSGLGLGEAK with glucoamylase (XP_007284956) of *Colletotrichum gloeosporioides* Nara gc5; and peptides DAALTMK and FNGAWGRPQR with glucoamylase (GAK68551) of *Pseudozyma antarctica*.

## Conclusions

All yeast species analyzed in this work showed the ability to assimilate SS or CMC, but none of them was able to carry out ethanol fermentation of these compounds. Relatively few yeast species showed an inducing effect of SS or CMC in the production of amylase or cellulase activity, respectively. For the majority of the analyzed yeast species, the two enzyme activities were higher when the culture medium was supplemented with glucose than with the corresponding substrate. The higher amylase activity values were obtained at pH 5.4 and 6.2; and at 30 and 37 °C, except for *Rh. glacialis* isolates, which showed high amylase activity at lower temperatures (10–22 °C). In general, the cellulase activity was high at pH values until 6.2 and from 22–37 °C; however, samples from *M. blollopis* showed high activity at lower temperatures (4–22 °C). According to peptide mass fingerprint analysis, the main extracellular protein of approximately 70 kDa in *Tetracladium sp*. culture supernatants with amylase activity indeed corresponds to a glucoamylase.

## Methods

### Yeast strains, media and culture conditions

The yeasts used in this work are listed in Table 1, and were previosly isolated by our group from soil samples of King George Island at the sub-Antarctic region [26]. The yeasts were routinely cultivated in YM medium (0.3 % yeast extract, 0.3 % malt extract, 0.5 % peptone) supplemented with 1 % glucose at their optimal temperature of growth and orbital shaking at 150 rpm. When specific carbon sources SS and CMC were used, these were supplemented to SD medium (0.1 % yeast nitrogen base, 0.05 % ammonium sulfate). In the case of semi-solid cultures, agar at 1.5 % was added to the medium.

**Table 1:** Yeast species used in this study

Species	Optimal growth (°C)	Cellulase activity	Amylase activity
*Cr. gastricus*	22	+	+
*Cr. gilvescens*	22	-	+
*Cr. victoiae*	22	+	-
*H. watticus*	22	+	+
*Cryptococcus* sp.	15	-	+
*Dioszegia* sp.	15	-	+
^*a*^ *Tetracladium* sp.	22	+	+
*D. fristingensis* (T9Df1)	15	-	+
*Le. fragaria*	22	+	-
*Leuconeurospora* sp. (T11Cd2)	15	-	+
*Leuconeurospora* sp. (T17Cd1)	22	+	-
*Leuconeurospora* sp. *(*T27Cd2)	22	+	-
*M. blollopis*	15	+	+
*M. gelida*	15	+	+
*M. robertii*	15	+	+
*Mrakia sp.*	15	+	+
*Rh. glacialis* (TRg8)	15	-	+
*Rh. glacialis* (T11Rs)	15	-	+
*W. anomalus*	22	+	-

The indicated enzyme activity reported in previous colony assays [26]. ^*a*^Formerly *D. fristingensis* (T11Df)

### Carbon source assimilation and fermentation tests

Yeasts were inoculated to reach an initial OD_600_ of 0.1 in flasks containing YNB medium supplemented with 0.125, 0.25, 0.5, 1 or 2 % w/v of SS or CMC. For each carbon source, 2 % was the maximum concentration that could be used due to an increase in viscosity of the medium. The flasks were incubated at 15 or 22 °C, and the growth was followed by measuring the OD_600_ of the culture. When the stationary phase was reached, 4 ml of the culture was centrifuged at 10,000 g for 10 min to determine the *c*ell *d*ry *w*eight (CDW) of the pellet dried at 100 °C for 24 h. The fermentation of both carbon sources was evaluated by using Durham test tubes [45] and by quantification of ethanol production using the Ethanol Assay Kit (Megazyme, Wicklow, Ireland) according to manufacturer’s instructions. All experiments were carried out in triplicates.

### Extraction of extracellular proteins and analysis

Extracellular proteins were obtained by centrifuging 100 ml of yeast cultures at 7,000 g for 10 min at 4 °C, and the obtained supernatants were filtered through a sterile 0.45-μm pore size polyvinylidene fluoride membrane (Millipore, Billerica, MA, USA). Then, ammonium sulfate was added to reach a final concentration of 80 % saturation and incubated on ice for 1 h followed by centrifugation at 10,000 g at 4 °C for 15 min. The pellet was suspended in 5 ml of Tris buffer (10 mM, pH 7.0) and dialyzed against the same buffer using a dialysis bag with a 10-kDa cut off. The total protein content was determined by the Bradford method (Coomassie Protein Assay Kit, Thermo Scientific, IL, USA) and analyzed on a 10 % SDS-polyacrilamide gel electrophoresis (SDS-PAGE) and then stained with silver nitrate (Merck KGaA, Darmstadt, Germany).

### Enzyme activity analysis

Protein samples were deposited into wells cut in YM-agar medium supplemented with 1 % of SS or CMC. The pH of media was adjusted with phosphate-citrate buffer and the plates were incubated at 4, 10, 15, 22, 30 or 37 °C. For amylase activity assays, the plates were flooded with 1 ml of iodine solution and positive activity was defined as a clear halo around the colony on a purple background [46]. For cellulose activity assays, the plates were flooded with 1 mg/ml of Congo red solution, which was poured off after 15 min and then the plates were flooded with 1 M NaCl for 15 min. Positive cellulase activity was defined as a clear halo around the colony on a red background [47]. Enzyme activities were measured as the distance in mm from the edge of the well to the halo normalized by total amount of protein of the sample.

### Peptide mass fingerprinting (PMF)

Processing and analysis of protein samples were performed at the Central Service for Experimental Research (Valencia, Spain). Samples were digested with sequencing grade trypsin (Madison, WI 53711 USA) as described previously [48]. The digestion reactions were stopped with TFA (1 % final concentration) and then concentrated to 20 μL by speed vacuum to analyze 5 μL by liquid chromatography and tandem mass spectrometry (LC–MS/MS). The obtained data were analyzed by Mascot, and only the results with a score greater than 54 (*P* < 0.05) were considered statistically significant [49].

### Availability of supporting data

All the supporting data are included as additional files.

## Additional file

Additional file 1:
**Enzyme activities in yeast extracellular protein samples.** Total proteins were obtained from cell-free supernatants of yeast species grown in YM media supplemented with SS (A) or CMC (B). The amylase (A) or cellulase (B) activity was evaluated by the well test assay at different pH and temperatures, and normalized by the total protein amount. The color gradient scale represents the degree of amylase activity from each yeast species from the lowest (L, black) to the highest (H, red) value. (PNG 1298 kb)

## Abbreviations

CMC: carboxymethyl cellulose
SS: soluble starch
