# Supplementary figures and images for: Screening and characterization of amylase and cellulase activities in psychrotolerant yeasts
Source: BMC Microbiol. 2016 Feb 19;16:21. doi: 10.1186/s12866-016-0640-8 (PMC4759947; doi:10.1186/s12866-016-0640-8)

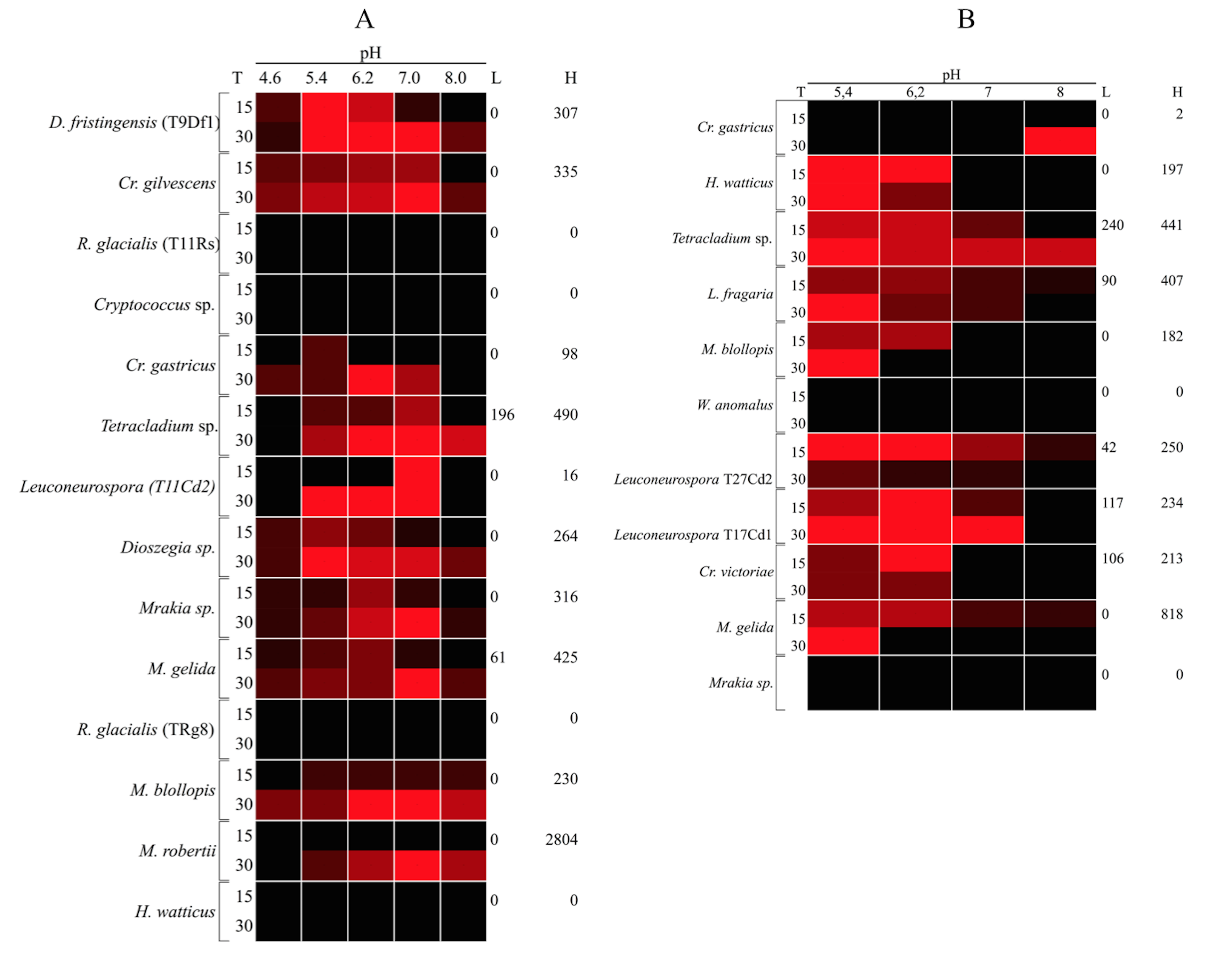

Supplement: Additional file 1: — Enzyme activities in yeast extracellular protein samples. Total proteins were obtained from cell-free supernatants of yeast species grown in YM media supplemented with SS (A) or CMC (B). The amylase (A) or cellulase (B) activity was evaluated by the well test assay at different pH and temperatures, and normalized by the total protein amount. The color gradient scale represents the degree of amylase activity from each yeast species from the lowest (L, black) to the highest (H, red) value. (PNG 1298 kb) [file 12866_2016_640_MOESM1_ESM.png]
